# First case report on molecular detection of *Trypanosoma lewisi* in an urban rat in Kelantan, Malaysia: An accidental finding

**DOI:** 10.5455/javar.2021.h531

**Published:** 2021-09-19

**Authors:** Intan Noor Aina Kamaruzaman, Hong Wei Ting, Muhammad Aiman Mohd Mokhtar, Yong Kai Yuan, Azim Wafiy Gulam Shah, Fathin Faahimaah Abdul Hamid, Che Wan Salma Che Wan Zalati, Nurshahirah Shaharulnizim, Mohd Farhan Hanif Reduan, Luqman Abu-Bakar

**Affiliations:** 1 Department of Paraclinical Studies, Faculty of Veterinary Medicine, Universiti Malaysia Kelantan, Kota Bharu, Kelantan, Malaysia; 2 Department of Clinical Studies, Faculty of Veterinary Medicine, Universiti Malaysia Kelantan, Kota Bharu, Kelantan, Malaysia

**Keywords:** Kelantan, polymerase chain reaction, rats, Trypanosoma lewisi

## Abstract

**Objective:**

This case report highlights the first detection of *Trypanosoma lewisi*, a blood protozoan parasite found in an urban rat in Kota Bharu, Kelantan.

**Materials and Methods:**

Rat trapping was carried out within the Kota Bharu vicinity near a local wet market. A total of 38 rats were captured and subjected to peripheral blood smearing using Giemsa stain. Positive rats were sent for histopathological analysis for the evaluation of the organ samples.

**Results:**

The presence of trypanosomes was found in one sample from a blood smear. This was connected to a histological lesion on kidney tissues, which revealed a high concentration of trypanosomes. Additionally, the positive sample was confirmed as *T. lewisi* based on molecular diagnosis via polymerase chain reaction and subsequent sequencing and phylogenetic analysis.

**Conclusions:**

This finding serves as a baseline for further surveillance on *T. lewisi* population among urban rats in Kelantan and possible zoonotic transmission to humans.

## Introduction

Trypanosomes are flagellated blood protozoa that can infect both warm-blooded and cold-blooded mammals [1]. It is a monophyletic genus of obligate intercellular parasites that induce trypanosomiasis in insects and vertebrates [2]. The organism is divided into two groups which are salivarian and stercorarian parasites. Both parasites are usually found in the digestive tract of the vector [3]. The parasite can cause severe diseases, such as human African trypanosomiasis (HAT) and Chagas disease in South and Central America. Not just in humans, this infection also can cause a decline in animal health, such as Surra and Nagana disease in horses [4]. Rodent species are primarily infected with *Trypanosoma lewisi*, which is commonly transmitted by several species of rat flea such as *Ceratophyllus fasciatus*, *Nosopsyllus fasciatus*, and *Xenopsylla cheopis*, respectively [5]. *T. lewisi* infection can be acquired by contact with feces of infected fleas or by ingestion of infected fleas [6]. This organism also can infect other mammals sporadically [7]. *Trypanosoma lewisi* can be found in urban and wild rats worldwide, especially in Asia [8,9]. Previously, it was thought that *T. lewisi* is a host-restricted parasite and does not possess a zoonotic risk to humans. However, several cases reported that the organism is an opportunistic pathogen that can cause infection to humans, including one fatal case [10–12].

Human infection with *T. lewisi* may show non-specific symptoms such as prolonged fever, appetite loss, and severe convulsion [11–13]. However, the effects of *T. lewisi* infestation on rodents are not well known. Therefore, knowing the zoonotic potential to humans with a close approximation to rodent population, here we report the first case of *T. lewisi* infection found in a wild rat in Kelantan, Malaysia. The finding is supported with blood smear results, histopathology, and confirmation by molecular diagnosis via polymerase chain reaction (PCR) and sequencing.

## Materials and Methods

### Ethical approval

This study was conducted after approval by the Animal Ethics Committee of Universiti Malaysia Kelantan with the reference no: UMK/FPV/ACUE/FYP/10/2021.

### Sample collection and identification of trypanosome

A total of 38 rats, 26 male and 12 female, were caught at random in the wet markets of Kota Bahru, Kelantan, Malaysia, to determine the incidence of leptospirosis and ectoparasites in the population of urban rats (manuscripts in preparation). All rats were euthanized using 2% (v/v) of carbon dioxide in a chamber (Dira Resources, Malaysia), and the blood was collected shortly via intracardiac puncture. A peripheral blood smear was conducted using Giemsa stain for blood routine examination. Several organs (liver, spleen, lungs, and kidneys) were harvested for subsequent diagnostic work-ups, including histopathology examination. 

### Detection of trypanosomes using universal trypanosome species (KIN) and T. lewisi-specific primer (TRYP1)

Suspected rats with trypanosomes were subjected to PCR to confirm the species using two sets of primers: KIN and TRYP1, respectively. The sequence of the primers is shown in Table 1. The thermocycler conditions for KIN primers are initial denaturation at 94°C for 3 min, denaturation at 94°C for 45 sec, annealing at 58°C for 3 min, and extension at 72°C for 3 min. The steps were repeated in 35 cycles and followed by further extension at 72°C for 3 min. For TRYP1 primer, the thermocycling parameters are initial denaturation at 94°C for 2 min, annealing at 55°C for 30 sec, and extension at 72°C for 30 sec, and the steps were repeated in 35 cycles followed by further extension step at 72°C for 10 min. The PCR products were visualized on a 2% (w/v) agarose gel stained with 1.0 μl Midori green and photographed under UV light. Sample with a positive result at PCR level was sequenced, and phylogenetic analysis using Molecular Evolutionary Genetics Analysis version 10.0 (PSU, USA) was constructed.

**Table 1.** List of trypanosome primers.

Primers	Detection	Sequence	Reference
KIN 1	Universal Trypanosoma	5ʹ-GCGTTCAAAGATTGGGCAAT-3ʹ	[14]
KIN 2		5ʹ-CGCCCGAAAGTTCACC-3ʹ	
TRYP1R	*T. lewisi*	5ʹ-GGA AGC CAA GTC ATC CAT CG-3ʹ	[3]
TRYP1S		5ʹ-CGT CCC TGC CAT TTG TAC ACA C-3ʹ	

## Results and Discussion

**Figure 1.**
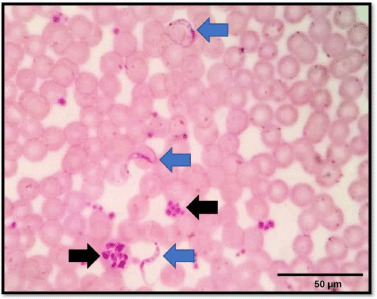
A micrograph showing the presence of multistage of *T. lewisi* in a thin blood smear of the infected rat (Case 1). Adult trypomastigotes are shown in blue arrows, characterized by a tadpole-like form with an undulating membrane, parabasal kinetoplast, oval nucleus, and a single flagellum. At the same time, epimastigotes (dividing stage of *Trypanosome*) are seen as an irregular shape with divided chromatin (shown in black arrows). Giemsa staining, 100×. Scale bar: 50 μm.

Out of 38 captured rats, 1 female brown rat (Case 1: *Rattus norvegicus*) had multiple polymorphisms of trypanosomes observed on a thin blood smear (Fig. 1). The organism was described as an individual, slender, tadpole-like form with a thin posterior end, oval kinetoplast, elongated nucleus, and a free flagellum known as adult trypomastigote. The appearance of adult trypomastigotes is suggestive for *T. lewisi* [15]. An unusual shape of trypanosomes with divided chromatins was found in the blood smear dividing stage epimastigotes. Both forms are usually present in acute infection in rats [16].

**Figure 2.**
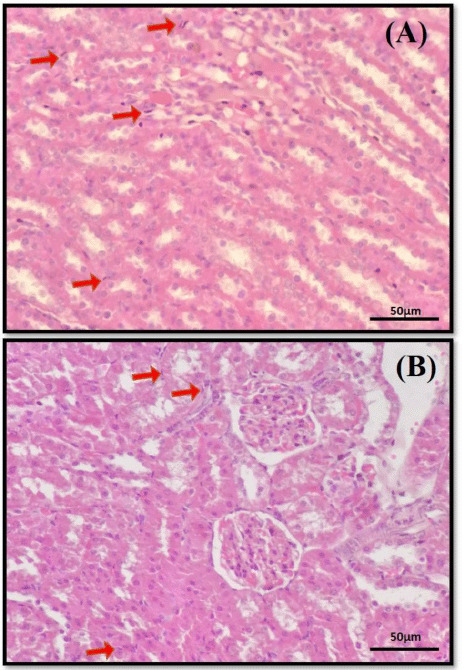
The histopathological finding of *T.lewisi* in this case. The abundance of *T. lewisi* epimastigotes can be seen in the kidney interstitium (A) and (B) indicated by red arrows. Hematoxylin and eosin staining, 40×. Scale bar: 50 μm.

**Figure 3.**
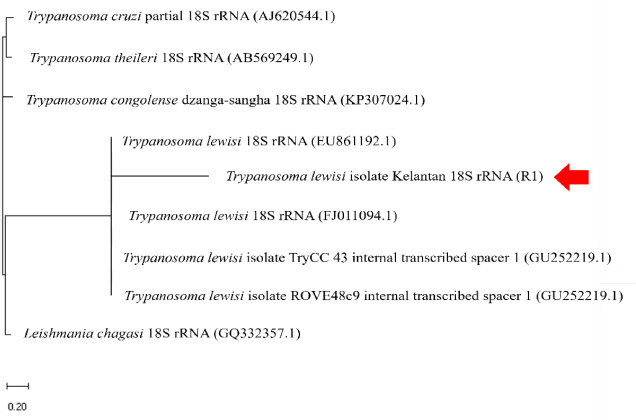
Phylogenetic analysis of *T. lewisi* in this case. The red arrow indicates a positive sample in this study.

Interestingly, in histopathology lesions, we found abundant epimastigotes in kidney tubules (Fig. 2). However, no evidence of inflammatory and degenerative tissue changes was observed in the kidney, and other organs showed normal tissue appearance (figures not shown). Furthermore, the sequencing and phylogenetic analysis of the PCR positive result was confirmative to *T. lewisi* (Fig. 3).

Although *T. lewisi* is a common parasitic protozoan found in rats worldwide, to our knowledge, this is the first evidence on *T. lewisi* found in a rodent reported in Kelantan, Malaysia. This rat was captured in a wet market within the vicinity of Kota Bharu. Previously, several studies described *Trypanosoma* spp. in urban rats in Malaysia at a low prevalence rate [17,18]. These studies reflected the presence of Trypanosome in rodents that live closer to the human setting, which may potentially expose the organism to the susceptible human host. The presence of *T. lewisi* polymorphism in the blood of rats in our study shows the establishment of an infective stage with the presence of both epimastigotes (development stage) and trypomastigotes (infectious stage; adult stage). This finding is interesting because epimastigotes are usually seen in the midgut of invertebrate vectors such as flies [19] and rarely in maintenance hosts [20]. It may be possible that stages of *T. lewisi* development occur in both maintenance host and invertebrate vectors compared to its closest relatives, such as *Trypanosoma cruzi* [21].

Apart from blood smear data, PCR and phylogenetic analyses (Fig. 3) further confirmed the evidence of *T. lewisi* presence in the rat. Although TRYP1 primers are specific for *T. lewisi*, the outcome from the PCR must be sequenced for confirmation as TRYP1 primers showed high cross-reactivity against other *Trypanosoma* species and other organisms related to rodents [3]. Nevertheless, both PCR are proven to be useful for screening of *T. lewisi* in rodents. 

The presence of epimastigotes in kidney cells revealed an exciting characteristic of the organism (Fig. 2). This showed that although *T. lewisi* is an obligate hemoparasitism, it is possible to migrate to multiple organs and exhibit a visceral form, as previously reported in *Trypanosoma evansi* [22]. The histological examination revealed *T. lewisi* in the interstitium of the kidney in our study. However, the parasites did not induce significant lesions to the organ. A study by Biswas et al. [23] showed that bandicoot rats affected with trypanosomiasis typically exhibited inflammatory, degenerative, and necrotic changes in multiple organs suggesting acute infection. These lesions were not seen in our case. However, different rat species may have varying levels of immunogenicity, and minor tissue alterations could imply that the brown rat is more tolerant to *T. lewisi* infection and hence acts as a principal reservoir or carrier for the parasite.

The occurrence of *T. lewisi* found in this study raises a public health concern among Kota Bharu residents, with its zoonotic potential to humans, possibly underreported. The organism is rapidly divided into mature parasites less than 24 h post-infection [24]. It possibly transmits vertically from mother to fetuses [25], further disseminating the organism within the rats’ population within a short time and to humans, subsequently. For this reason, a rodent surveillance program is warranted to estimate the prevalence of *T. lewisi* in the wild rat’s population in Kota Bharu and alert the health authorities on its effect on humans. Additionally, molecular surveys on the type of vectors carrying *T. lewisi* are indicated to evaluate the tendency of transmission between rodent-borne trypanosomes and humans. Such studies can help provide essential information on the diversity of these vectors toward designing an effective preventive strategy to eliminate the possible infection.

## Conclusion

*Trypanosoma lewisi* is a common parasitic infection in wild rodents which potentially transmit to humans. This is the first case report of trypanosomiasis in rats in the Malaysian state of Kelantan. Further surveillance is needed to ascertain the prevalence of *T. lewisi* in rodent populations to assess the risk of human infection.

## List of Abbreviations

KIN: Universal trypanosome species; PCR: polymerase chain reaction; *T. lewisi*: *Trypanosome lewisi*; TRYP1: *T. lewisi*-specific primers.

## References

[1] Rasoanoro M, Ramasindrazana B, Goodman SM, Rajerison M, Randrianarivelojosia M (2019). A review of *Trypanosoma* species known from Malagasy vertebrates. Malagasy Nat.

[2] Hong XK, Zhang X, Fusco OA, Lan YG, Lun ZR, Lai DH (2017). PCR-based identification of *Trypanosoma lewisi* and *Trypanosoma musculi* using maxicircle kinetoplast DNA. Acta Trop.

[3] Milocco C, Kamyingkird K, Desquesnes M, Jittapalapong S, Herbreteau V, Chaval Y (2011). Molecular demonstration of *Trypanosoma evansi* and *Trypanosoma lewisi* DNA in wild rodents from Cambodia, Lao PDR and Thailand. Transbound Emerg Dis.

[4] Magri A, Galuppi R, Fioravanti M (2021). Autochthonous *Trypanosoma *spp. in European mammals: a brief journey amongst the neglected trypanosomes. Pathogens.

[5] Lee CM, Armstrong E (2008). Rodent trypanosomiasis: a comparison between *Trypanosoma lewisi* and *Trypanosoma musculi.* Encyclopedia of Entomology.

[6] Gao JM, Truc P, Desquesnes M, Vincendeau P, Courtois P, Zhang X (2018). A preliminary serological study of *Trypanosoma evansi* and *Trypanosoma lewisi* in a Chinese human population. Agric Nat Resour.

[7] Ortiz PA, Garcia HA, Lima L, da Silva FM, Campaner M, Pereira CL (2018). Diagnosis and genetic analysis of the worldwide distributed Rattus-borne *Trypanosoma (Herpetosoma) lewisi* and its allied species in blood and fleas of rodents. Infect Genet Evol.

[8] Rayat CS, Vashista RK (2014). Wild rats as reservoir of *Trypanosoma lewisi* in Northwest India. Austin J Pathol Lab Med.

[9] Pumhom P, Morand S, Tran A, Jittapalapong S, Desquesnes M (2015). Trypanosoma from rodents as potential source of infection in human-shaped landscapes of South-East Asia. Vet Parasitol.

[10] Sarataphan N, Vongpakorn M, Nuansrichay B, Autarkool N, Keowkarnkah T, Rodtian P (2005). Diagnosis of a *Trypanosoma lewisi*-like (Herpetosoma) infection in a sick infant from Thailand. J Med Microbiol.

[11] Shah I, Ali US, Andankar P, Joshi RR (2011). Trypanosomiasis in an infant from India. J Vector Borne Dis.

[12] Verma A, Manchanda S, Kumar N, Sharma A, Goel M (2011). Banerjee PS, et al. Case report: *Trypanosoma lewisi* or *T. lewisi*-like infection in a 37-day-old Indian infant. Am J Trop Med Hyg.

[13] Howie S, Guy M, Fleming L, Bailey W, Noyes H, Faye JA (2006). A Gambian infant with fever and an unexpected blood film. PLoS Med.

[14] Mwandiringana E, Gori E, Nyengerai T, Chidzwondo F (2012). Polymerase chain reaction (PCR) detection of mixed trypanosome infection and blood meal origin in field-captured tsetse flies from Zambia. Afr J Biotechnol.

[15] Desquesnes M, Ravel S, Cuny G (2002). PCR identification of *Trypanosoma lewisi*, a common parasite of laboratory rats. Kinetoplastid Biol Dis.

[16] de Sousa MA (2014). On opportunist infections by *Trypanosoma lewisi* in humans and its differential diagnosis from *T. cruzi* and *T. rangeli*. Parasitol Res.

[17] Alias SN, Sahimin N, Edah MA, Mohd-Zain SN (2014). Epidemiology of blood parasitic infections in the urban rat population in peninsular Malaysia. Trop Biomed.

[18] Siti Shafiyyah CO, Jamaiah I, Rohela M, Lau YL, Siti Aminah F (2012). Prevalence of intestinal and blood parasites among wild rats in Kuala Lumpur, Malaysia. Trop Biomed.

[19] Barrias E, Reignault LC, de Souza W (2019). How does the main infective stage of *T. cruzi* enter and avoid degradation in host cells? A description of the pathways and organelles involved on these processes. Biology of *Trypanosoma cruzi*. IntechOpen.

[20] Misra KK, Roy S, Choudhury A (2016). Biology of *Trypanosoma* (Trypanozoon) *evansi *in experimental heterologous mammalian hosts. J Parasit Dis.

[21] Onyekwelu KC (2016). Life cycle of *Trypanosoma cruzi *in the invertebrate and the vertebrate hosts. IntechOpen.

[22] Ghaffar MA, El-Melegy M, Afifi AF, El-Aswad BEDW, El-Kady N, Atia AFI (2017). The histopathological effects of *Trypanosoma evansi *on experimentally infected mice. Menoufia Med J.

[23] Biswas D, Choudhury A, Misra KK (2001). Histopathology of* Trypanosoma* (Trypanozoon) *evansi *infection in Bandicoot rat. I. visceral organs. Exp Parasitol.

[24] Zhang X, Li SJ, Li Z, He CY, Hide G, Lai DH (2019). Cell cycle and cleavage events during *in vitro* cultivation of bloodstream forms of *Trypanosoma lewisi,* a zoonotic pathogen. Cell Cycle.

[25] Cencig S, Coltel N, Truyens C, Carlier Y (2013). Fertility, gestation outcome and parasite congenital transmissibility in mice infected with TcI, TcII and TcVI genotypes of *Trypanosoma cruzi*. PLoS Negl Trop Dis.

